# Factors associated with place of death for terminal cancer patients who wished to die at home

**DOI:** 10.1097/MD.0000000000030756

**Published:** 2022-09-30

**Authors:** Eun Jee Lee, Na-Ri Lee

**Affiliations:** a College of Nursing, Research Institute of Nursing Science, Jeonbuk National University, Jeonju, Republic of Korea; b Division of Hematology and Oncology, Department of Internal Medicine, Jeonbuk National University Hospital, Jeonbuk National University Medical School, Jeonju, Republic of Korea; c Research Institute of Clinical Medicine, Jeonbuk National University, Biomedical Research Institute of Jeonbuk National University Hospital, Jeonju, Republic of Korea.

**Keywords:** cancer, caregiver, hospices, place of death, preference, quality of dying

## Abstract

A patient’s desired place of death is an important indicator of the quality of dying. The purpose of this study was to investigate the actual places of death of terminal cancer patients who wished to die at home and the factors affecting their actual place of death. A retrospective survey was used to analyze the medical records of 143 terminal cancer patients who wanted to die at home among a population of 168 patients who used a home hospice care service more than once between March 2016 and December 2019. Patients who wanted to die at home represented 85.1% of the total study population (143 patients). Of these, 31.5% and 68.5% were home and hospital deaths, respectively. Factors associated with the actual place of death of patients who desired to die at home were marital status (odds ratio [OR] = 2.57, confidence interval [CI]: 1.08–6.13), the patient’s status at the time of their enrollment in a home hospice care service (OR = 3.30, CI: 1.56–7.02), and the primary caregiver’s relationship with the patient (OR = 2.52, CI: 1.12–5.66). Most terminal cancer patients studied did not die in their preferred place. Support from policies and hospice professionals is needed to decrease caregiver burden and help patients die wherever they want. Consequently, quality of end-of-life care can be improved.

## 1. Introduction

Being able to die in the place preferred by the patient is an important indicator of the quality of palliative care.^[1]^ The preferred place of death may vary depending on one’s cultural background^[2]^: for example, in Asian cultures, many terminal cancer patients prefer to die at home.^[3,4]^ The reason most Koreans wish to die at home is it is a familiar space where they can pass away while being surrounded by family members.^[5]^ Conversely, some of terminal cancer patients prefer to die in a hospital, hoping to receive treatment until the end, or to reduce their families’ burden of care.^[4]^

In a previous study conducted in South Korea (“Korea” hereafter), among the patients who preferred to receive care at home, only 17.53% received such care.^[6]^ According to Korean national death statistics, 77.1% of terminal cancer patients die in hospitals, with 13.8% dying at home,^[7]^ with the proportion of hospital deaths increasing over time.^[5]^

To guarantee the desired quality of death for terminal cancer patients, patients should be able to die in their chosen location, and healthcare professionals should help these patients.^[8]^

Thus, this study aimed to provide fundamental data for future hospice care programs by identifying the status of the actual places of death of terminal cancer patients, along with the factors associated with their inability to die at home.

This study was specifically conducted to identify the factors associated with the place of death of terminal cancer patients who preferred to die at home. The specific purposes of this study are as follows:

1. Identify the preferred and actual place of death of home-based hospice-palliative care patients.
2. Identify differences in general characteristics, disease-related characteristics, and primary caregivers’ characteristics according to the place of death of home-based hospice-palliative care patients.
3. Identify the factors associated with the actual place of death of terminal cancer patients who preferred to die at home.

## 2. Methods

### 2.1. Study design

A retrospective study was conducted to analyze electronic medical records and identify factors associated with the place of death of patients who preferred to die at home.

### 2.2. Participants and data collection

Among the 187 terminal cancer patients who registered for home-based hospice-palliative care services at least once at J University Hospital between March 1, 2016, and December 16, 2019, 143 met the following criteria and were selected as subjects for this study:

1. Inclusion criteria
  Patients who died from terminal cancer
  Patients who preferred to die at home
2. Exclusion criteria
  - Patients who preferred to die in a hospital
  - Patients whose actual place of passing was unknown
  - Patients who were alive at the time of data collection

**Figure 1.**
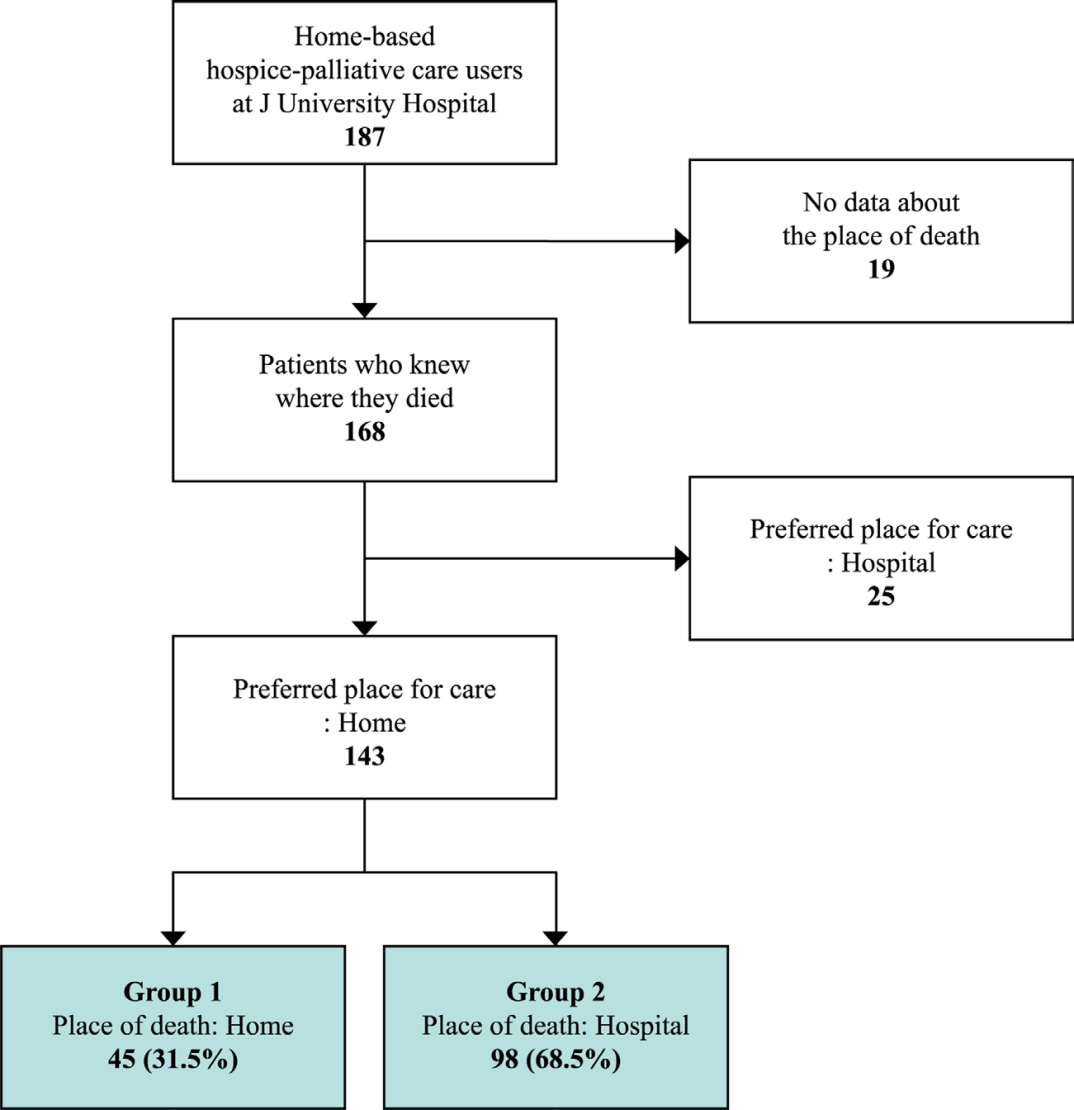
Flowchart of study participants.

As shown in Figure 1, of the 143 study participants who preferred to die at home, those classified in Group 1 died at home, while those in Group 2 died in a hospital. This study was conducted after receiving an exemption from the institutional review board of the Jeonbuk National University Hospital (CUH 2020-10-018).

### 2.3. Research variables

1. *Preferred and actual places of death*

The preferred place of death was collected from hospice nurses’ initial evaluation records, and the actual place of death was collected from patients’ electronic medical records. The place of death was classified as either home (Group 1) or hospital (Group 2).

2. *General characteristics*

Data concerning gender, age (at the time of initial registration for home-based hospice care services), marital status, type of medical insurance, and religion were collected.

3. *Disease-related characteristics*

The following data were also collected: insights concerning terminal status, mental status, place of care before hospice care request, pain at the time of home-based hospice registration and 1 week after registration (using the numerical rating scale [NRS] pain scale: 1–10 points), status at the time of registration, and the period from terminal diagnosis to death.

4. *Characteristics of primary caregivers*

Regarding the primary caregiver, data on gender, age, relationship with the patient, and whether he/she lived with the patient were collected. The number of family members living with the patients was also collected.

### 2.4. Data analysis methods

The data were analyzed using SPSS 26. Frequencies and percentages were used to describe patients’ preferences regarding their place of death and the actual place of death for those receiving home-based hospice care services. Chi-square tests and *t* tests were conducted to compare the differences between general characteristics, disease-related characteristics, caregiver characteristics, and place of death. Logistic regression analysis was also performed to investigate the factors affecting the place of death.

## 3. Results

### 3.1. Subjects’ preferences regarding their preferred and actual places of death

Table 1 shows the preferred and actual places of death for the 168 study participants. The number of patients who wanted to die at hospital was 25 (14.9%), whereas 143 (85.1%) patients wished to die at home. Among the patients who wanted to die at home, 45 (31.5%) were able to do so, while 98 (68.5%) of these patients died in hospital.

**Table 1:** Participants’ preferences regarding their preferred and actual places of death.

Actual place of death	Preferred place of death	
	Home (n = 143, 85.1%)	Hospital (n = 25, 14.9%)
	n (%)	n (%)
Home	45 (31.5): Group 1	0 (0)
Hospital	98 (68.5): Group 2	25 (100)

### 3.2. Differences in patients’ characteristics by group

Table 2 shows the differences between the 2 groups according to patients’ and caregivers’ characteristics.

**Table 2:** Differences in patients’ characteristics by group.

Variables	Categories	Total (n = 143)	Group 1 (n = 45, 31.5%)	Group 2 (n = 98, 68.5%)	*t* or *x*^2^	*P*
		n (%) or M (SD)	n (%) or M (SD)	n (%) or M (SD)		
**Patients**						
Gender	Male	77 (53.8)	26 (57.8)	51 (52.0)	0.408	.523
	Female	66 (46.2)	19 (42.2)	47 (48.0)		
Age		68.91 (13.89)	69.09 (14.02)	68.83 (13.91)	0.104	.917
Marital status	Married	100 (69.9)	37 (82.2)	63 (64.3)	4.718	.030
	Divorced/widowed/separated/married	43 (30.1)	8 (17.8)	35 (35.7)		
Type of insurance	Health insurance	136 (95.1)	44 (97.8)	92 (93.9)	1.008	.433*
	Medical benefit	7 (4.9)	1 (2.2)	6 (6.1)		
Religion	Protestant	59 (41.3)	13 (28.9)	46 (46.9)	4.232	.375
	Buddhist	17 (11.9)	6 (13.3)	11 (11.2)		
	Catholic	21 (14.7)	8 (17.8)	13 (13.3)		
	Others	5 (3.5)	2 (4.4)	3 (3.1)		
	None	41 (28.7)	16 (35.6)	25 (25.5)		
Having insight of terminal status	Yes	134 (93.7)	44 (97.8)	90 (91.8)	1.846	.273*
	No	9 (6.3)	1 (2.2)	8 (8.2)		
Mental status	Alert	131 (91.6)	39 (86.7)	92 (93.9)	2.086	.194*
	Drowsiness, stupor, coma	12 (8.4)	6 (13.3)	6 (6.1)		
Place of care before request	Home	116 (81.1)	32 (71.1)	84 (85.7)	4.340	.127*
	Facility (nursing home)	14 (9.8)	7 (15.6)	7 (7.1)		
	Hospitals (including the acute care ward/hospice ward)	13 (9.1)	6 (13.3)	7 (7.1)		
Pain at the time of home-based hospice registration		2.38 (1.94)	2.67 (1.99)	2.26 (1.92)	1.178	.241
Pain 1 week after registration			1.67 (1.12)	1.81 (1.37)	−0.543	.588
Status at the time of registration	Stable	99 (69.2)	23 (51.1)	76 (77.6)	10.121	.001
	Unstable or dying	44 (30.8)	22 (48.9)	22 (22.4)		
Period from terminal diagnosis to death (d)		89.25 (158.74)	51.76 (58.97)	109.09 (189.18)	−1.981	.011
**Primary caregiver**						
Gender	Male	39 (27.3)	15 (34.1)	24 (24.7)	1.322	.250
	Female	102 (71.3)	29 (65.9)	73 (75.3)		
Age		56.67 (15.00)	54.57 (14.73)	57.67 (15.11)	−1.130	.261
Relationship with patient	Spouse	71 (49.7)	17 (37.8)	54 (55.1)	5.482	.065
	Parents	9 (6.3)	2 (4.4)	7 (7.1)		
	Children	43 (30.1)	19 (42.2)	24 (24.5)		
	Other (sibling, daughter-in-law/son-in-law, caregiver, etc)	20 (14.0)	7 (15.6)	13 (13.3)		
Lives with primary caregiver	Yes	127 (88.8)	37 (82.2)	90 (92.8)	3.629	.078
	No	15 (10.5)	8 (17.8)	7 (7.2)		
Number of household members		1.93 (1.23)	2.04 (1.33)	1.88 (1.18)	0.755	.451

M = mean, SD = standard deviation

* Fisher exact test.

Results of the chi-square test showed that marital status (*x*^2^ = 4.718, *P* = .030) and status at the time of registration for home hospice care (*x*^2^ = 10.121, *P* = .001) were found to be significant variables affecting outcomes. The *t* test revealed that the period from terminal diagnosis to death (*t* = −1.981, *P* = .011) was significant.

### 3.3. Factors influencing the place of death for patients who preferred to die at home

To identify the factors affecting the place of death for patients who preferred to die at home, a logistic regression analysis was performed using variables that showed statistically significant differences via the univariate analysis (marital status, status at the time of registration for home hospice service, the period from a terminal diagnosis to death), the primary caregiver’s relationship with the patient, and cohabitation of the primary caregiver and patient. The explanatory power of the final model was statistically significant, with a Nagelkerke *R*^2^ of 0.413 (−2 log likelihood 103.88, *P* < .001). The hospital death rate was significantly higher for divorced/bereaved/separated/unmarried patients than for those who were married (odds ratio [OR] = 2.57, confidence interval [CI]: 1.08–6.13), and for stable patients than for unstable or dying patients (OR = 3.30, CI: 1.56–7.02). Furthermore, the hospital death rate was higher when the primary caregiver was the patient’s spouse, rather than the patient’s child (OR = 2.52, CI: 1.12–6.66) (see Table 3).

**Table 3:** Factors associated with hospital deaths of patients who preferred to die at home.

Factors	Categories	OR (95% CI)	*P*
**Patients**			
Marital status	Divorced, bereaved, separated, unmarried	2.57 (1.08–6.13)	<.001
Status at the time of registration	Stable	3.30 (1.56–7.02)	.001
Period from terminal diagnosis to death (d)		1.01 (1.00–1.01)	.426
**Primary caregiver**			
Relationship with Patient	Spouse	2.52 (1.12–5.66)	<.001
	Parent	2.77 (0.52–14.91)	.491
Lives with the primary caregiver	No	0.36 (0.12–1.06)	.890
−2 log likelihood		103.88	
Hosmer & Lemeshow *x*^2^ (*p*)		3.97 (0.411)	
Nagelkerke *R*^2^		0.413	

CI = confidence interval, OR = odds ratio, Reference: marital status (married), status at the time of registration (unstable or dying), relationship with patient (child), lives with the primary caregiver (yes).

## 4. Discussion

A retrospective survey was conducted using the electronic medical records of patients who used home hospice services more than once to determine the factors affecting the place of death of terminal cancer patients who prefer to die at home.

The preference for a patient’s place of death may differ by country or culture.^[9,10]^ In this study, the number of patients who preferred to die at home was much larger than that of those who preferred to die in a hospital. Unlike the patients’ preferred place of death, the actual place of death for more than two-thirds of the patients was a hospital, with the proportion continuing to increase. A similar result was reported in a previous study,^[11]^ wherein the author considered that this mismatch between the preferred place of death and actual place of death could be related to the patients’ cause of death. In Europe, however, the percentages of home and hospital deaths were similar; meanwhile, hospital deaths have been decreasing while home deaths have been increasing, differing from the results reported elsewhere.^[8,12,13]^

In this study, the factors influencing the place of death of patients who wanted to die at home were marital status, status at the time of registration for home hospice services, and the relationship between the primary caregiver and patient.

First, the percentage of hospital deaths was higher among divorced, separated, bereaved, and unmarried than among married patients. Data regarding marital status are the most basic in determining the degree of patients’ social support, and in many studies, the enduring marital relationships of terminally ill patients were important predictors of death at home.^[9,12,13]^ Married patients were more likely to die at home because they had more human resources/potential caregivers available than divorced/separated/widowed/unmarried patients. Therefore, if patients who prefer to die at home do not have human resources available to them (in order to take care of and support them), hospice professionals should be considered appropriate caregivers.

Second, patients’ status at the time of registration for home hospice care services was found to be a factor that significantly influenced their decision regarding the place of their death. In this study, patient status was categorized as stable, unstable, or dying. A stable condition refers to a state in which physical and mental symptoms are controlled without requiring a change to the care plan, whereas an unstable condition refers to a state in which intensive monitoring and adjustment of care plans are required given the patient’s insufficient control over physical and mental symptoms. A dying period indicates that the patient is expected to die within a few days; therefore, preparation is required to control the symptoms associated with dying.^[14]^

In this study, patients in a stable condition were more likely to die in a hospital than those in either an unstable condition or a state of death. In Asian cultures, terminal cancer patients with severely limited functions had a higher rate of home deaths than those in Western cultures,^[5,15–17]^ indicating that patients with severe functional limitations perceived their disease as being in an advanced state and feeling that death was imminent. Accordingly, help from caregivers could easily be accepted by patients dying at home.^[9]^ In the same context, for patients in a stable condition, the caregiver’s fatigue or burden may be high given an extended period of home care; thus, hospital deaths may be high. In this study, the period between terminal diagnosis and death did not affect the place of death according to the logistic regression analysis; however, the univariate analysis revealed that the period was significantly shorter for patients who died at home than for those who died in hospitals. However, further studies are required to obtain more reliable results.

Third, the probability of patients dying in a hospital was higher when the spouse was the primary caregiver than when the child filled that role. This result has been repeatedly reported in many previous studies.^[9,18,19]^ A possible reason is the burden on the spouse caring for the patient. In Asian cultures, when determining the place of death, there is a tendency to consider the burden of the caregiver and the degree of care needed by the patient, rather than individual values.^[20]^ Patients in households consisting of patients and spouses were more likely to die in a hospital than patients in households consisting of multiple members.^[21,22]^ Additionally, when the spouse was the primary caregiver and the caregiving burden was large, the spouse’s own rest, health, and stress level tended to improve after the patient’s death.^[23]^ Therefore, if the spouse was the primary caregiver and had a heavy caregiving burden, the possibility of the patient’s hospital death was greater. Another possibility is that Korean families tend to be more involved in patients’ treatment decisions than the patients themselves and believe that it is their duty to ensure that patients receive all the care they can provide. Thus, when the primary caregiver is a spouse, the spouse may proactively strive to ensure that the patient receives cancer treatment and assists with symptom management.^[3,24–26]^

This study had several limitations. Given the retrospective methodology of analyzing the medical records of the deceased, it was not possible to extensively analyze the factors affecting decisions regarding the place of death for terminal cancer patients who wanted to die at home. However, this study was the first to compare cases of dying at home in light of the preference of patients to do so and cases of dying in a hospital despite the preference to die at home. Therefore, the results of this study can be used as basic data for appropriately allocating limited hospice resources and as the basis for further research aimed at respecting a patient’s preference regarding their place of death.

## 5. Conclusions

In conclusion, the findings of this study revealed that although 85.1% of the Korean terminal cancer patients (who were subjects of this study) preferred to die at home, only 31.5% actually died at home. A patient who was divorced/separated/widowed/unmarried, who was stable at the time of registration for hospice care, and whose primary caregiver was his/her spouse, generally did not die at home; rather, death in a hospital was characteristic. Therefore, the government should implement policies that encourage hospice professionals to regularly evaluate primary caregivers’ needs and risk factors, thus preventing burnout. Additionally, there should be a policy that helps identify terminally ill cancer patients with no caregivers early and assist them in choosing a health care proxy. Only with such policy support will terminal cancer patients receive appropriate end-of-life care services and die whenever they wish.

## Author contributions

**Data curation:** Na-Ri Lee.

**Methodology:** Eun Jee Lee.

**Resources:** Na-Ri Lee.

**Supervision:** Na-Ri Lee.

**Writing – original draft:** Eun Jee Lee.

**Writing – review & editing:** Na-Ri Lee.
